# Fractures-luxations de Monteggia chez l’enfant: étude rétrospective de 40 cas dans le Département d’Orthopédie, Centre Hôpital Universitaire Habib Bourguiba de Sfax, Tunisie

**DOI:** 10.11604/pamj.2022.43.25.35191

**Published:** 2022-09-16

**Authors:** Nizar Sahnoun, Maissa Ben Mabrouk, Mourad Aoui, Yosr Hentati, Bilel Feriani, Zoubaier Ellouz, Hassib Keskes

**Affiliations:** 1 Service de Chirurgie Orthopédique et Traumatologique, Centre Hospitalier Universitaire Habib Bourguiba, Sfax, Tunisie,; 2 Département de Médecine de Famille, Faculté de Médecine de Sfax, Sfax, Tunisie,; 3 Service de Radiodiagnostic et d´Imagerie Médicale, Centre Hospitalier Universitaire Hedi Chaker, Sfax, Tunisie

**Keywords:** Fracture, luxation, cubitus, tête radiale, enfant, consolidation, Fracture, dislocation, elbow, radial head, child, consolidation

## Abstract

Les fractures luxations de Monteggia représentent une entité rare en traumatologie infantile, elles représentent essentiellement un problème de délai de prise en charge et un réel problème pronostique. L'objectif de ce travail est de décrire les caractéristiques épidémiologiques de cette fracture, d´évaluer nos résultats fonctionnels et anatomiques. Nous rapportons une série de 40 cas de fracture Monteggia chez l´enfant colligés et traités au centre hospitalo-universitaire Habib Bourguiba de Sfax sur une période de 17 ans, allant de janvier 1998 au janvier 2015. Nous avons recensé les données épidémiologiques de notre population ainsi que les types des fractures luxations selon les classifications radiologiques Bado. Pour l´évaluation fonctionnelle, le choix du traitement était basé sur le type de fracture. Nous avons choisi les scores de l´équipe du P. Rigault et le score de Kim. L´âge moyen de notre population était d 8 ans. 20 cas des fractures étaient classés Bado I et 12 cas étaient Bado III. Le délai de prise en charge était moins de 24h dans 82% des cas. Le traitement chirurgical était réalisé dans 28 cas, et nos résultats fonctionnels ont été jugés bons dans 30 cas. Nos résultats étaient satisfaisants grâce à notre délai de prise en charge considéré précoce par rapport à ceux de la littérature.

## Abstract (en)

Monteggia's fracture-dislocation is rare in pediatric traumatology and pose a problem of elapsed time before management and of prognosis. The purpose of this study was to describe the epidemiological features of this fracture and to assess functional and anatomical results. We report a series of 40 cases of Monteggia fracture in children whose data were collected and treated at the Habib Bourguiba University Hospital Center in Sfax over a period of 17 years, from January 1998 to January 2015. We listed the epidemiological data of our population as well as the types of fracture dislocations according to the Bado radiological classifications. For functional assessment, the choice of treatment was based on the type of fracture. P. Rigault’s score and Kim's score were used. The average age of our study population was 8 years; 20 cases of fractures were classified as Bado I and 12 cases as Bado III. The time elapsed before treatment was less than 24 hours in 82% of cases. Surgical treatment was performed in 28 cases, and functional results were judged to be good in 30 cases. Our results were satisfactory due to the short time elapsed before patient management compared to literature data.

## Introduction

Les fractures de Monteggia ont été décrites initialement par Giovanni Batista Monteggia en 1814, qui associe souvent une fracture diaphysaire de l´ulna avec une luxation de la tête radiale [1]. Elles représentent une entité rare en traumatologie infantile [2]. L´une des nombreuses complications de la lésion aiguë de Monteggia est le retard de diagnostic, en particulier chez les enfants. La prise en charge des lésions de Monteggia invétérées non diagnostiquées en urgence représente un scénario clinique difficile ce qui rend la prise en charge plus difficile [3]. Le but de cette étude est de décrire nos résultats fonctionnels et anatomiques des fractures de Monteggia chez l´enfant.

## Méthodes

**Cadre de l´étude:** nous rapportons une série de 40 cas de fracture Monteggia chez l´enfant colligés et traités au Centre Hôpital-Universitaire Habib Bourguiba de Sfax sur une période de 17 ans, allant de janvier 1998 au janvier 2015.

**Collecte des données:** la collecte des cas de fractures de Monteggia s´est fait à partir des registres médicaux. Des fiches d´exploitation préétablies ont été remplies regroupant les paramètres épidémiologiques, cliniques, thérapeutiques et évolutifs, ainsi qu´à la convocation des patients pour évaluer les résultats à long terme.

**Sources de données/mesures:** nous avons recensé l´âge, le sexe, les antécédents des patients locaux et généraux, les circonstances de survenue, le mécanisme lésionnel et la date de survenu de la lésion. Tous les patients ont été explorés par un bilan radiographique standard complet comportant une radiographie du coude, de l´avant-bras et du poignet de face et de profil. A Travers ce bilan les lésions observées sont classées selon des classifications radio-cliniques afin de pourvoir bien évaluer les données anatomopathologiques de la lésion. Nous avons adopté la classification de Bado qui est basée sur le sens de luxation de la tête radiale et décrit ainsi 4 types de lésion de Monteggia [4-6]. Le type I correspond à une fracture ulnaire proximale avec angulation antérieure avec une luxation antérieure de la tête radiale. Le type II correspond à une fracture de la diaphyse cubitale avec une angulation postérieure avec une luxation de la tête radiale de même postérieure ou postéro-latérale. Le type III, il s´agit d´une fracture de la métaphyse cubitus avec une luxation de la tête radiale latérale ou antéro-latérale. Enfin le type IV correspond à une fracture du tiers proximal des deux os ulnaire et radial avec luxation antérieure de la tête radiale. Les résultats fonctionnels ont été évalués sur des critères cliniques et radiologiques bons, moyens ou mauvais. Nous avons adopté 2 scores, les critères proposés par l´équipe du P. Rigault et celle de J.P Padovani (Tableau 1) et le score de Kim (Tableau 2, Tableau 3).

**Tableau 1:** critères d’évaluation des résultats selon P. Rigault et de J.P Padovani

Critères d'évaluation			Note
**Aspects cliniques**	Prono-suppination	Normale	2
		Diminuée de ¼	1
		Très diminuée	0
	Flexion-extension	≥ 120°	2
		≤ 90°	1
		< 90°	0
**Aspects radiologiques**	Radius	Tête normale en place	2
		Tête anormale en place	1
		Tête luxée	0
	Ulna	Normal	2
		Petit cal vicieux	1
		Gros cal vicieux	0

**Tableau 2:** score de Kim de performance du coude

Critère		Score
**Déformation**	Absente	25
	Mineure	15
	Majeure	0
**Douleur**	Absente	25
	Intermittente sans limitation d'activité	15
	Persistante avec limitation d'activité	0
**Secteur de mobilité du coude (flexion-extension + prono-supination)**	>250°	25
	Entre 250° et 200°	15
	<200°	0
**Fonction du coude**	Sans problème	25
	Difficulté	15
	Impotence totale	0

**Tableau 3:** évaluation clinique selon le score de Kim

Score de Kim	> 90	89-75	74-60	< 60
Evaluation	Excellent	Bon	Juste	Pauvre

**Les variables quantitatives et qualitatives:** les données qualitatives ont été décrites en nombre. Les données quantitatives ont été décrites par des moyennes.

## Résultats

**Participants:** dans notre série, la fracture de Monteggia a touché plus fréquemment les enfants scolarisés surtout la tranche d´âge entre 1 et 18 ans, avec un âge moyen de 8 ans. Une prédominance masculine a été observée: 28 garçons et 12 filles.

**Figure 1:**
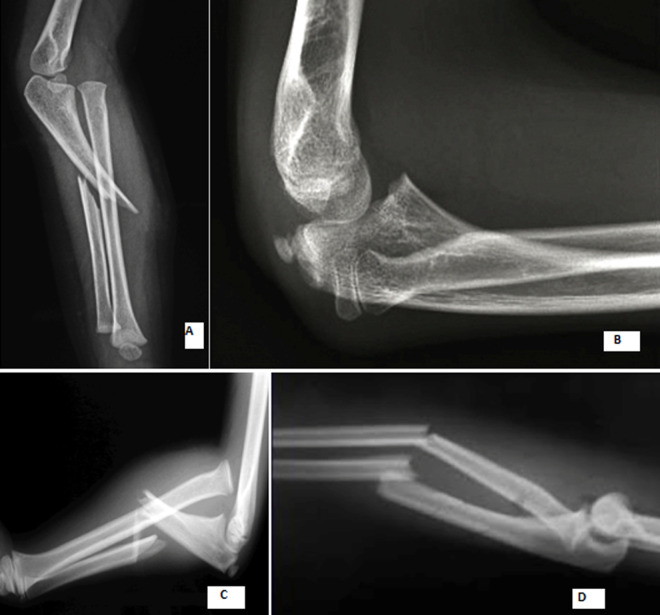
type anatomopathologique selon Bado: A) Bado I; B) Bado II; C) Bado III; D) Bado IV

**Données descriptives:** les traumatismes étaient le plus souvent directs et de faible énergie faisant suite à des accidents domestiques ou scolaires. Le côté gauche a été touché dans 62,5% cas. L´analyse anatomopathologique de nos cas a montré une prédominance des fractures diaphysaires de l´ulna jonction tiers moyen tiers supérieure, observées dans 24 cas, associées à une luxation antérieure de la tête radiale dans la majorité des cas. Les fractures métaphyso-épiphysaires retrouvées dans 14 cas, étaient toutes à l´origine d´une luxation latérale ou antéro-latérale de la tête radiale. Le type de fracture ulnaire était sous forme de déformation plastique dans 1 cas, déplacée dans 27 cas, non déplacée dans 5 cas et en bois vert dans 6 cas. La luxation de la tête radiale était antérieure dans 19 cas, postérieure dans 6 cas et latérale dans 15 cas. D´après la classification de Bado, nous avons observé 20 cas de type Bado I, 7 cas de type Bado II, 12 cas de type Bado III et enfin 1 seul cas de type Bado IV (Figure 1).

La fracture Monteggia s´est associée à un décollement du col du radius dans 2 cas. Dans 33 cas de notre série le diagnostic et la prise en charge était faite dans les 24 premières heures, 7 cas ont été traités tardivement à J4, J6, J10 et J15 post traumatique. Le traitement orthopédique a été appliqué dans 12 cas qui consisté à une réduction sous anesthésie générale avec immobilisation plâtrée par un plâtre brachio-antérobrachial. Ce plâtre a été maintenu pendant une durée moyenne de 6 semaines, avec des extrêmes allant de 4 à 9 semaines. La consolidation a été obtenue en un délai de 4 à 9 semaines, moyennement 5 semaines. A l´aide du contrôle radiologique hebdomadaire, qui a été fait pendant les trois premières semaines, un seul cas de reluxation de la tête radiale après déplacement secondaire du foyer ulnaire a été observé au quinzième jour de la réduction initiale, repris chirurgicalement. Le traitement chirurgical a été indiqué dans 28 cas.

**Figure 2:**
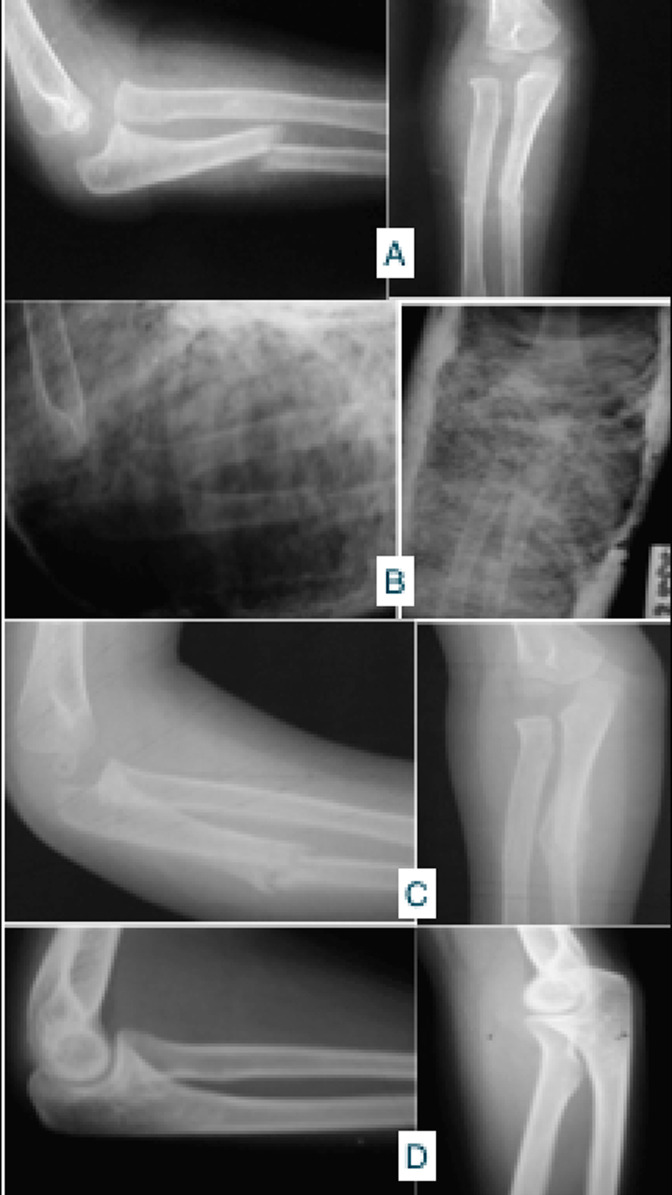
résultats après consolidation: A) radiographie face et profil de la fracture; B) radiographie face et profil après plâtre brachio anté brachial; C) résultat à un mois après un traitement orthopédique par plâtre brachio-antéro-brachial; D) résultat anatomo-radiologique à 10 ans de recul

**Figure 3:**
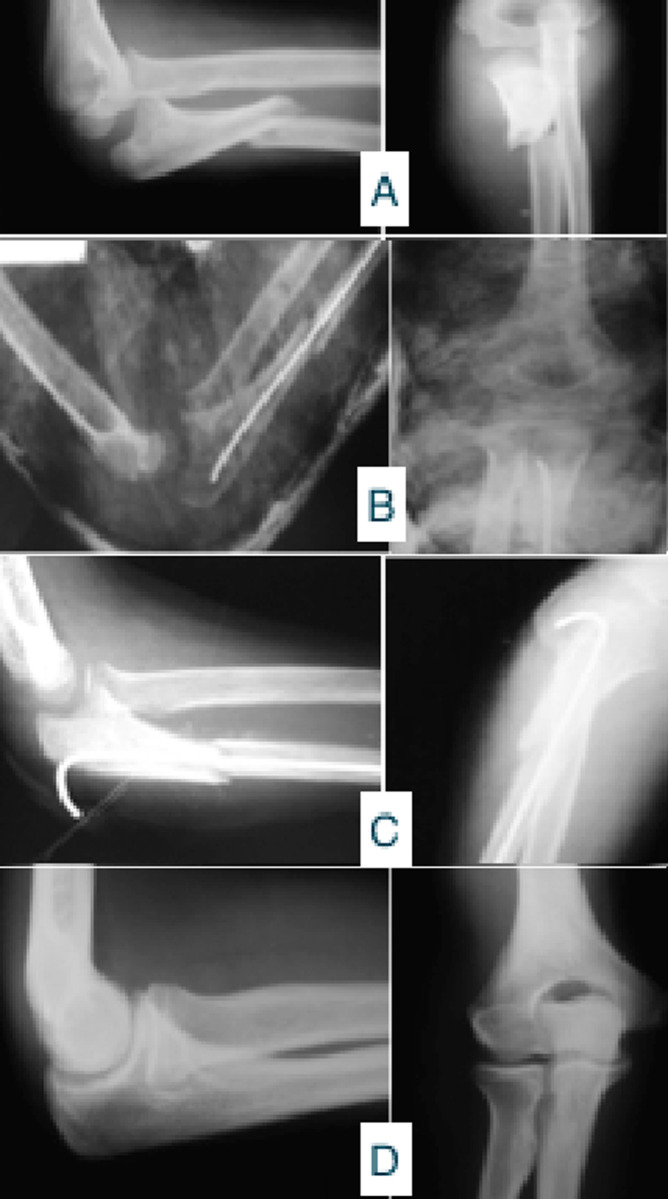
résultats après consolidation: A) radiographie face et profil de la fracture; B) résultat après embrochage; C) résultat à un mois après embrochage; D) résultat anatomo-radiologique à 10 ans de recul

La synthèse ulnaire a été pratiquée par embrochage centromédullaire dans 17 cas, par plaque vissée dans 8 cas, 2 cas embrochage-haubanage et un haubanage de l´olécrane dans un cas. La réduction de la tête radiale a suivi généralement celle de l´ulna dans tous les cas sauf dans 3 cas (un cas classé Bado 4 et deux cas fracture Monteggia invétérée), où nous étions obligés d´aborder cette dernière, de la réduire et de la stabiliser à ciel ouvert par une broche radio-ulnaire proximale. L´immobilisation après le traitement chirurgical a été assurée soit par une attelle brachio-antébrachiale chez 7 patients ou un plâtre brachio-tébrachial dans 21 cas. La consolidation osseuse a été obtenue dans un délai moyen de 8 semaines, avec des extrêmes allant de 4 à 12 semaines (Figure 2, Figure 3).

Dans notre série, presque la totalité de nos patients n´ont pas eu de complications précoces ni en per ni en post opératoire sauf un seul cas qui a présenté une paralysie du nerf radial en préopératoire et qui a récupéré spontanément au bout de 5 mois, et un autre cas qui a présenté un syndrome de Volkmann au quatrième jour post opératoire ayant nécessité l´ablation du plâtre, avec bonne évolution. La totalité de nos patients n´ont pas eu de complications tardives. En se référant aux critères d´appréciation de Padovani ainsi le score de Kim nos résultats ont été jugés bons dans 29 cas et moyens dans les 11 autres cas. Nos meilleurs résultats ont été observés chez les enfants d´âge scolaire, diagnostiqués précocement et pris en charge en urgence, quelle que soit la méthode thérapeutique adoptée.

## Discussion

La fracture de Monteggia n´est pas une lésion fréquente en milieu de traumatologie infantile, seulement 2 à 6,5% des fractures luxation du coude [2]. Elle est d´une incidence annuelle moyenne de 2,6 cas par an [7,8]. Cette fracture est plutôt l´apanage des enfants de tranche d´âge entre 4 et 10 ans [2,7,9]. Les enfants dans notre population avaient un âge moyen de 8,8 ans. Une prédominance masculine est de même notée. Ces résultats s´expliquent par un comportement de turbulence des garçons à cet âge [2,10,11]. Le mécanisme le plus fréquent était un traumatisme direct, souvent un coup direct appliqué à l´avant-bras lors d´un classique mouvement de protection « Fracture du mauvais garçon » [12-14]. Le diagnostic positif de la lésion de Monteggia récente repose principalement sur la radiologie standard de l´avant-bras qui doit être complet et rigoureux dans son exécution et dans sa lecture, prenant les deux articulations coude et poignet avec les deux incidences face et profil, car les lésions ne sont pas toutes visibles sur un seul cliché, d´où l´importance de faire deux incidences afin d´éviter de passer à côté cette lésion qui facilement peut être inaperçue [15].

La fracture de l´ulna et la luxation de la tête radiale, en précisant son type et son importance, sont généralement faciles à voir. La subluxation de la tête radiale reste difficile à reconnaître, surtout en population pédiatrique où les os du coude sont incomplètement ossifiés [16]. Nous avons eu recours à la construction de la ligne de Stören, qui est l´axe passant par l´extrémité supérieure du radius et le milieu du noyau d´ossification condylien quelques soient l´incidence et le degré de la flexion du coude, pour pouvoir mettre en évidence cette luxation de la tête radiale [14]. La déviation de cette ligne radio-capitulaire confirme la luxation ou la subluxation de la tête radiale [17]. Ces éléments radiologiques observés, aussi bien mentionnées dans la littérature, sont importants pour les classifications de Bado et de Trillat et qui nous a guidé par conséquent dans la prise en charge ultérieure [18]. Le diagnostic de la fracture-luxation Monteggia à temps est important à fin d´éviter les lésions Monteggia invétérées. Il s´agit d´une urgence diagnostique et thérapeutique [19].

Le principe du traitement de la fracture Monteggia, quel que soit orthopédique ou chirurgical, repose sur la stabilisation de la tête radiale et la réduction de la fracture ulnaire. Nous avons préconisé le traitement orthopédique souvent de principe pour la fracture Monteggia récente de l´enfant [20]. En cas de lésions anciennes dépassant les 3 semaines, le résultat a été souvent un échec dans la plupart des cas. Même dans la littérature, les études cliniques observées avec des lésions Monteggia invétérées ont montré que le traitement orthopédique n´est pas supérieur dans ce cas [21]. Dans la littérature, le traitement de la lésion Monteggia était chirurgical dans 99,4% des cas [14,22]. Il est le traitement de choix en cas de fracture ulnaire instable, d´instabilité de la tête radiale après réduction de la fracture ulnaire, de fracture ouverte, dans le cadre des polytraumatismes, ou pour rattraper un traitement orthopédique échoué. Le délai moyen entre le traumatisme et la chirurgie varie entre les 4 semaines et 10 ans. Bouyala [21] a constaté au cours d´une étude faite sur 12 luxations persistantes de la tête radiale dont le délai avant l´intervention varie de 2 mois à 13 mois, qu´à partir de 2 ans, les résultats étaient moins bons, mais qu´un bénéfice est toujours possible même après 2 ans, et qu´il ne faut jamais renoncer à intervenir.

Delpont *et al*. [22] ont constaté que l´intervention de Bouyala donne de bons résultats à long terme, dans les lésions Bado type I, quel que soit l´âge, en cas de prise en charge avant 1 an. Rahbek *et al*. [23] et Stoll *et al*. [24] ont insisté aussi sur la prise en charge précoce de la lésion de Monteggia. Ceci montre ainsi l´importance de la précocité de la prise en charge de la fracture Monteggia. Nous avons utilisé différentes voies d'abord celles aussi discutée dans la littérature. Il s´agit de 4 voies d´abord : la voie postéro-latérale, soit externe [25] ou interne [26], la voie externe de Kocher [27] et la voie antéro-externe de Henry. L´ostéosynthèse de l´ulna était assurée par plusieurs moyens chirurgicaux [28].

Le traitement par embrochage centromédullaire trouve sa place devant l´échec du traitement orthopédique pour les fractures Monteggia qui présente une instabilité voire une irréductibilité [29]. Dans notre série, les fractures Monteggia traitées par embrochage cubital sont celles itératives à un traitement orthopédique, soit 60,8% des patients. Une grande partie des séries dans la littérature ont aussi rapporté un chiffre élevé des patients traités par embrochage centromédullaire. Le brochage centromédullaire chez l´enfant constitue la technique d´ostéosynthèse de choix respectant les cartilages de croissance et la vascularisation périostée. Il peut être réalisé en percutané après une réduction anatomique du foyer ulnaire. Le traitement chirurgical par plaque vissée sont peu utilisés chez l´enfant voir même abandonnés [30]. Dans notre série, 28,6% des patients ont bénéficié d´ostéosynthèse par plaque vissée qui est comparable à la littérature, M. Letts [11] rapporte 27% et la plupart du reste le nombre des patients ayant subis cette intervention ne dépasse pas les 10%. L´haubanage est surtout utilisé devant les fractures de l´extrémité supérieure de l´ulna. Elle se fait par deux broches avec un cerclage en huit de chiffre par un fil en acier ou en vicryl.

Dès la consolidation, les broches devraient être enlevés évitant toute migration des broches et tout conflit avec la peau qui est source d´infection [28]. Nos résultats globaux sont comparables à ceux publiés dans la majorité des séries. Dans la littérature, d´excellents et de bons résultats globaux du traitement des fractures récentes de Monteggia ont été observées dans 70 et 100% des cas. Ceci témoigne de la relative bénignité de cette fracture traitée précocement et convenablement, contrastant avec l´importance des éléments lésés et la gravité potentielle de la négligence de cette fracture- luxation. A la lumière de notre série, et après une revue de la littérature, nous avons essayé de dégager certains facteurs qui ont un effet pronostique sur l´évolution de ce type de fracture qui sont essentiellement le délai de prise en charge et le type anatomopathologique de la fracture selon les classifications de Bado et Trillat.

## Conclusion

Les fractures Monteggia de l´enfant sont des lésions simples de la traumatologie pédiatrique si diagnostiquées et traitée correctement en urgence. Toute la gravité découle de leur méconnaissance. Ceci impose une sensibilisation des jeunes cliniciens et des urgentistes pour une rigueur d´observation chez tout enfant traumatisé du membre supérieur, une exploration radiologique complète du coude et de l´avant-bras, une conduite thérapeutique appropriée avec une surveillance radio -clinique régulière.

### Etat des connaissances sur le sujet

- Les fractures Monteggia sont des entités rares en la traumatologie pédiatrique;
- Les fractures Monteggia chez l´enfant posent un problème diagnostic et thérapeutique;
- Les fractures Monteggia chez l´enfant posent un problème pronostique à cause des retards de diagnostic et des complications des lésions invétérées.

### Contribution de notre étude à la connaissance

- Le traitement des fractures Monteggia chez l'enfant peut être orthopédique ou chirurgical;
- La compréhension de la fracture est un temps capital dans la planification thérapeutique;
- La prise en charge à temps donne de bons résultats fonctionnels.
